# Current Status and Challenges of Planetary Health Education: Protocol for a Scoping Review

**DOI:** 10.2196/70910

**Published:** 2025-08-26

**Authors:** Yayoi Shoji, Kana Suzuki, Masushi Kohta, Nao Tamai

**Affiliations:** 1 Public Health Unit Global Cooperation Institute for Sustainable Cities Yokohama City University Yokohama Japan; 2 Institute of Tropical Medicine Nagasaki University Nagasaki Japan; 3 Research Center for Implementation Nursing Science Initiative Fujita Health University Toyoake Japan; 4 Department of Nursing Graduate School of Medicine Yokohama City University Yokohama Japan

**Keywords:** public health education, climate change and health, educational frameworks, competency-based education, interdisciplinary education

## Abstract

**Background:**

The impacts of climate change, such as air pollution and abnormal weather, are becoming more apparent and threatening to human health. In response, the concept of planetary health was proposed in 2015 based on the recognition that human and earth health are closely related and that the general public needs to improve the health and sustainability of the ecosystems through a comprehensive approach. As economic development and human longevity progress, the burden on the earth’s environment will increase; however, systematic methods for education and planetary health practices have not yet been established.

**Objective:**

This study aims to clarify the current status and challenges of planetary health–related education. Specifically, it seeks to identify and analyze both existing educational programs that have already been implemented and evaluated, as well as those that have not yet been systematically assessed, providing insights for future curriculum development.

**Methods:**

A scoping review based on PRISMA (Preferred Reporting Items for Systematic Reviews and Meta-Analyses) guidelines will be conducted using the Population-Concept-Context framework. Without a defined target period, original papers and conference proceedings published in PubMed, CINAHL, EMBASE, Web of Science, Scopus, Google Scholar, and the Cochrane Central Register of Controlled Trials will be searched, and the findings will be disseminated through peer-reviewed publications and conference presentations. Studies with descriptions of planetary health education are included, while review papers and studies published in languages other than English or Japanese are excluded.

**Results:**

As of August 2025, 28 studies had been selected. Data collection was carried out between May 2024 and July 2025. Preliminary data analysis will be conducted again in September 2025. The results are currently being prepared for publication and are expected to be published by October 2025.

**Conclusions:**

This study will identify the current status and challenges of planetary health education. The findings will contribute to developing future curricula that promote ecosystem health and sustainability.

**International Registered Report Identifier (IRRID):**

DERR1-10.2196/70910

## Introduction

Climate change poses a significant threat to human health and well-being, and the United States Centers for Disease Control and Prevention has identified air pollution, extreme weather events, heat waves, water quality deterioration, and increased allergens as potential consequences [1]. In 2015, The Rockefeller Foundation and The Lancet proposed the concept of planetary health. This concept emphasizes the inseparable relationship between human and planetary health and provides a holistic approach to comprehensively improving and maintaining the health of all ecosystems [2,3]. It is important to distinguish planetary health from related concepts such as “One Health” and “sustainability education.” Although One Health primarily focuses on zoonotic diseases and the interconnected health of humans, animals, and ecosystems, and sustainability education broadly addresses environmental justice and resource management, planetary health emphasizes the interdependence of human health and the state of the earth’s natural systems. This concept is not only concerned with protecting the global environment but also includes recognition of inequalities and injustices. In particular, many low-income communities tend to be more vulnerable to the severe impacts of climate change [4], and the need to address both environmental inequality and social injustice simultaneously is emphasized. International organizations such as the Planetary Health Alliance are working to understand the interplay between the environment and health and to promote education and training to address these issues [5].

The importance of planetary health education is increasingly recognized; yet, significant gaps remain in its global implementation. Previous studies have reported that the adaptation to planetary health education policies has been slow, with a lack of faculty expertise and an emphasis on treatment rather than prevention, which has become a significant barrier to curriculum integration [6]. Furthermore, there is limited information on how cultural, socioeconomic, and political factors should be integrated into planetary health education curricula.

In particular, challenges specific to planetary health education include the absence of standardized competencies, the fragmented incorporation of content into existing health or environmental curricula, and a lack of interdisciplinary collaboration in curriculum development and delivery. These challenges hinder the development of cohesive educational strategies needed to prepare students as future leaders in addressing climate-related health issues. Against this background, how to integrate planetary health education into broad university-wide curricula as well as specialized programs such as medical, science, and teacher education is recognized as a critical challenge moving forward.

Therefore, this study aims to clarify the current status and challenges of planetary health–related education. We hypothesize that planetary health education at universities is not yet systematically organized, and there is considerable variation in the educational content, objectives, and teaching methods among programs. Specifically, we seek to identify both educational programs that have already been implemented and evaluated as well as those that have not yet been systematically assessed while also examining the educational objectives, teaching methods, and resources. This study will focus on how planetary health is taught to university students, educators, and health care professionals, all of whom have the potential to become future leaders and key contributors to societal well-being. The study will further examine the differences and challenges in planetary health education across general education courses and specialized programs at the university level. By doing so, this study aims to explore the effectiveness of these educational approaches and identify strategies for improving and advancing planetary health education. The focus of this study is on university and professional education curricula, with secondary education excluded from the scope. The reason for excluding secondary education (middle and high school levels) is that the specialized content of planetary health is more commonly taught at the university level. Education at the secondary education level tends to focus on developing basic awareness of environmental issues, and the concept of planetary health is often not fully developed at these stages. Therefore, this study focuses on university and professional education.

The results of this study will be shared with the author’s affiliated educational institution and collaborating researchers in various countries, and these results will be used in the design and improvement of planetary health education programs at each institution.

## Methods

### Study Design

This scoping review will investigate how planetary health education is being implemented. The research protocol follows the PRISMA-ScR (Preferred Reporting Items for Systematic Reviews and Meta-Analyses Extension for Scoping Reviews) [7] guidelines.

### Identifying the Research Question

This study will aim to clarify how the concept of planetary health is incorporated into educational programs. Based on our findings, we plan to develop and implement educational programs in the future. The concept of planetary health used in this study was jointly introduced by The Rockefeller Foundation and The Lancet. Our research question is: what are the current status and challenges of planetary health education? For scoping reviews, the Population, Concept, Context framework will be used [7]. In this study, the components defined are as follows.

Population: students, educators, and health care professionals.

Concept: the current state and challenges of planetary health education, its delivery methods, content, program design, and curriculum.

Context: geographic location (country, region), educational settings (university, public educational institutions, online platforms), cultural or political background, and specific socioeconomic context in which education is conducted.

### Identifying Relevant Studies

To identify potentially relevant and gray literature, databases such as CINAHL, Scopus, EMBASE, Ichushi-Web, PubMed, and OpenGrey will be searched without setting a specific time frame. All the references will be imported into Rayyan and Microsoft Excel for deduplication and screening. The PubMed search strategy will be adopted as the primary search approach, and the search strategies for other databases will align with this strategy to ensure consistency across platforms. The preliminary search string will be as follows: (“Planetary health”[title/abstract]) AND (education[MeSH terms])

We will include all relevant studies published up to March 2025 to ensure comprehensive topic coverage. No lower limit on the publication date was set. Studies with descriptions of planetary health education are included, while review papers and studies published in languages other than English or Japanese are excluded.

### Study Selection

**Figure 1:**
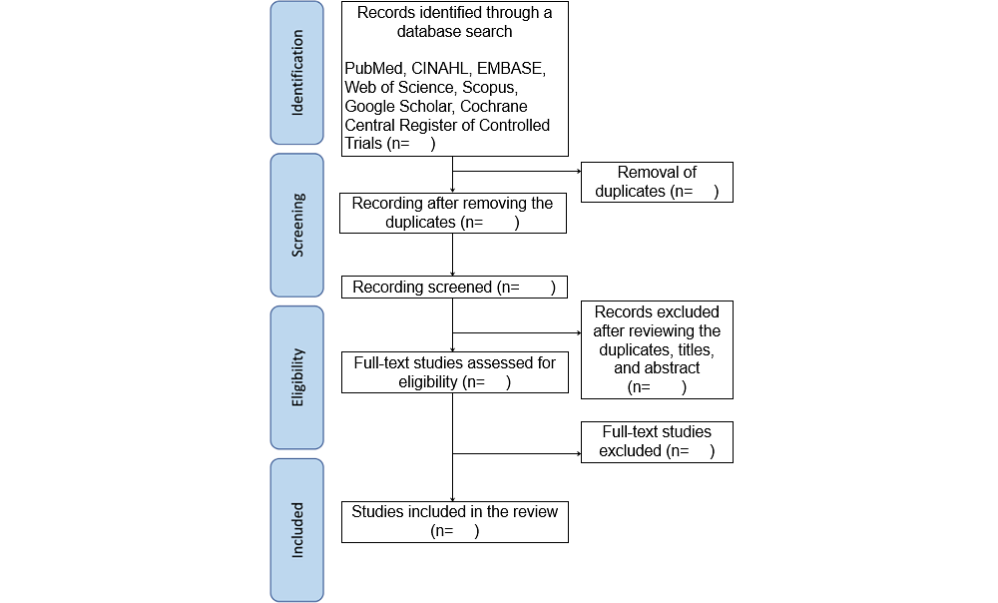
Flowchart of the study selection process.

The studies included in this scoping review will address the integration of planetary health into educational programs, specifically targeting university students and professionals, excluding middle and high school students. Eligible literature will consist of original research studies, gray literature, and conference proceedings. Reviews, letters, comments, and books will be excluded. No restrictions will be applied regarding the study design. Study selection will be conducted using Rayyan and Excel (Microsoft Excel, version 16.35). Two reviewers will independently screen the studies based on titles and abstracts during the initial screening by using the eligibility criteria. The eligibility criteria will be assessed by 2 reviewers, and any discrepancies will be resolved through discussion or third-party adjudication. Subsequently, 2 reviewers will independently review the full texts of the studies that meet the eligibility criteria based on their titles and abstracts. In cases where the 2 researchers are uncertain of their judgments, a third researcher will be consulted to provide a definitive decision. The reasons for excluding studies after full-text review will be documented. The study selection process is summarized in a PRISMA flow diagram (Figure 1).

### Charting the Data

The data extraction process involves Rayyan and Microsoft Excel. To provide an overview of the trends in the selected literature resulting from the searches, we will create a table based on Garrard’s [8] metric approach. The selected papers will be listed chronologically and organized into 8 topics: author(s), publication year, country, objectives, study design, participants, survey items, and results. In order to consider the influence of the cultural, socioeconomic, and political factors on planetary health education, we will also examine in detail how the differences in the educational environments across regions as well as the social and cultural backgrounds of each region are reflected in the educational programs. Specifically, we will analyze how each country’s educational systems and policies, region-specific social issues, and cultural values influence the introduction and implementation of planetary health education. By comparing and examining these factors, we aim to strengthen the applicability of this study in different educational settings. When conducting the analysis, we will carefully address the variations in the terminology, limited data availability, and differences in the educational systems across regions. Additionally, when comparing the results, we will carefully consider the educational environments and the policies specific to each region. To strengthen the evaluation of the impact of planetary health education, this study will also discuss how the effectiveness of the programs can be assessed. Potential evaluation criteria will include measures such as students’ retention of knowledge, behavioral changes, and the adoption of planetary health curricula by educational institutions. By considering these outcome-based evaluations, we aim to gain a more comprehensive understanding of the educational impact of planetary health initiatives. These aspects will be included in the review to provide a clearer picture of the outcomes associated with planetary health education.

### Result Collation, Summarization, and Reporting

Given that the methodologies and the study populations of the various studies included in this scoping review will differ, the analysis of the results will depend on the type of data collected. Further, the identified studies will be analyzed using qualitative methods. Where feasible, the results will be categorized within the domains proposed by the Planetary Health Education Framework [9], which are outlined in 5 foundational areas. Based on the 5 foundational areas of this framework, the selected studies will be categorized and compared, clearly identifying the educational outcomes and challenges within each area to evaluate the effectiveness of planetary health education. Furthermore, by using the framework, we aim to ensure consistency in data interpretation and to systematically understand the educational impacts across different regions and educational systems.

### Ethics and Dissemination

As this scoping review will not require any intervention or population recruitment, approval from a research ethics board was not necessary. The results of this scoping review will be disseminated through peer-reviewed publications and conference presentations. This will allow us to make recommendations for the future development of educational programs related to planetary health in universities.

## Results

As of August 2025, 28 studies had been selected. Data collection was carried out between May 2024 and July 2025. Preliminary data analysis will be conducted again in September 2025. The results are currently being prepared for publication and are expected to be published by October 2025.

## Discussion

The strength of this scoping review lies in its ability to identify current gaps and to provide a foundation for the development of curricula that integrate human health and planetary health. These findings are expected to contribute to the construction of sustainable educational strategies that emphasize both ecosystem and human health. However, this study is limited to published research and cannot cover the curricula of all educational institutions. As a result, it may not be possible to fully clarify the current state and challenges of planetary health education.

Moreover, we acknowledge that the scope of the published literature may introduce risks of institutional and publication biases as well as the dominance of Western-centric frameworks influencing educational content. Such biases may limit the representation of diverse educational models and regional perspectives, particularly from non-Western countries where planetary health education may be emerging differently. In addition, this study limited the literature search to publications in Japanese and English. This decision was made because the research team is proficient in these languages, allowing for accurate and reliable analysis without the risk of misunderstandings or loss of nuance through translation. In the future, as collaborators from countries such as Indonesia, Vietnam, Thailand, and Malaysia join the team, we plan to expand the scope to include literature published in other languages. Recognizing these limitations, we intend to address these issues in future studies by collaborating with researchers from diverse geographical and cultural backgrounds to expand the scope and the inclusivity of the educational frameworks reviewed.

With economic development and human longevity progressing, modern society is placing an unprecedented burden on the environment. The United Nations Development Program’s Planetary Pressures–adjusted Human Development Index, introduced in the 2020 Human Development Report, is one attempt to quantitatively assess this global challenge [10]. Although Europe leads the Planetary Pressures–adjusted Human Development Index rankings, Southeast Asian countries have reported a lack of sufficient environmental and health initiatives. Climate change performance indicators evaluate international policies across 4 categories: greenhouse gas emissions, renewable energy, energy use, and climate policies. Although no country meets all the criteria, the European Union has received high ratings, while Southeast Asian nations such as Thailand, Vietnam, and Indonesia have demonstrated low performance, with Japan and Malaysia also rated very low. The average temperature in this region has risen by 0.14 °C to 0.20 °C per decade since the 1960s, with projections of further temperature increases and more frequent heatwaves [10].

These challenges underscore the importance of comprehensive planetary health education. Although various models have existed, such as the “One Health” programs focused on zoonotic diseases and sustainability programs addressing food security and environmental justice [11], there are limited programs specifically focused on planetary health. According to previous research, more than 95 universities and organizations are promoting education and research related to planetary health [12]. However, many of these efforts remain confined to specific academic disciplines or fields, and there are still challenges in developing comprehensive curricula that adopt an interdisciplinary approach. Furthermore, integrating planetary health into existing curricula has been reported to face barriers such as a lack of educator expertise and insufficient understanding within educational institutions [6]. The current status and challenges of planetary health education presented in this scoping review can help deepen our understanding of the obstacles educators face as well as provide guidance for institutions to implement effective curriculum reforms. Moreover, this study is expected to contribute to building a foundation for more effective educational strategies by emphasizing the importance of interdisciplinary approaches and offering insights into how to integrate planetary health into educational systems. Additionally, it is anticipated that this research will lay the groundwork for future educational programs to better adapt to regional needs and incorporate the concept of planetary health comprehensively into educational settings.

## Abbreviations

PRISMA: Preferred Reporting Items for Systematic Reviews and Meta-Analyses
PRISMA-ScR: Preferred Reporting Items for Systematic Reviews and Meta-Analyses Extension for Scoping Reviews
